# Wounds beyond bars: reflecting on the urgent need for psychological support and interventions in the aftermath of Syria’s regime collapse

**DOI:** 10.1192/bji.2025.10047

**Published:** 2025-11

**Authors:** Obada Al-leimon, Mohammed Saadeh, Abdulrahman Ghaibeh, Khalid Elzamzamy

**Affiliations:** 1 School of Medicine, The University of Jordan, Amman, Jordan; 2 School of Medicine, The University of Jordan, Amman, Jordan. Email: mohammedsaadeh26@gmail.com; 3 Department of Psychiatry, Faculty of Medicine, Damascus University, Damascus, Syria; 4 Department of Psychiatry and Behavioral Sciences, Johns Hopkins University School of Medicine, Baltimore, Maryland, USA

**Keywords:** Syria, post-traumatic stress disorder, mental health crisis, torture, prisoners

## Abstract

Decades of systemic oppression in Syria, from the 1963 state of emergency to the 2011–2024 conflict, have caused widespread psychological devastation. Arbitrary imprisonment, torture and sexual violence have been systematically weaponised. Following the fall of the Syrian regime in December 2024, freed political prisoners face severe mental health challenges due to years of inhumane conditions and trauma. This paper emphasises the urgent need for specialised mental health interventions and outlines evidence-based pathways to healing. A coordinated, multi-tiered response, integrating local and international efforts, is essential to prioritise mental health aid, restore dignity and support survivors in rebuilding their futures.

Human rights violations in Syria are deeply rooted, with a long and troubling history that stretches back as far as Syrians can remember. Imprisonment and unfair trials have been prevalent since the enforcement of the state of emergency in 1963.^1^ This decades-long pattern of society-wide abuse has created a persistent and institutionalised reality of torture and horror.

Since the Syrian conflict outbreak in 2011, a vast humanitarian crisis has engulfed a large portion of the Syrian people. Countless individuals have been displaced, injured, maimed or lost family members. These tragedies, exacerbated by chaos and poverty rates, have had a profoundly impact on Syrians’ mental health. The psychological toll has been evident, with numerous cases of post-traumatic stress disorder (PTSD) and severe depression reported.^2^

The Syrian Network for Human Rights estimates that between March 2011 and August 2024, 157 634 Syrians were arbitrarily imprisoned or had forcibly disappeared, including 5274 children and 10 221 women, with 15 393 deaths due to torture, 86.7% of them by the regime forces.^3^ It is also believed that at least 100 000 of them were killed and buried in mass graves.^4^ Amid this tragedy, the issue of prisoners and forcibly disappeared individuals came to the forefront in December 2024, when they were liberated after the fall of the Syrian regime.

## The trauma of imprisonment

Prisoners lived in inhumane conditions, including overcrowded, unlit rooms or dark underground solitary cells in poorly maintained and unsanitary facilities. They were subjected to torture, starvation, deprivation of medical care, sexual violence and psychological abuse. This included being forced to imitate animal sounds, threats of assault or rape against them or their family members, and the psychological distress caused by torture, verbal abuse or solitary confinement.^5^ Widespread diseases, such as tuberculosis, coupled with torture and summary executions, claimed countless lives.^5^ In some cases, bodies were left among the living for days before removal.^5^ These inhumane conditions were not exclusive to a single facility or security institution, despite the notoriety of prisons like Sednaya and Tadmor.

The psychological impact on recently freed prisoners is profound, with many psychiatric conditions emerging as a critical concern. Many of them are survivors of rape, military combat, captivity and politically motivated persecution. Syrian prisoners experienced all of these, directly or through witnessing or being informed of such acts. Prolonged captivity and repeated exposure to traumatic events may lead survivors to experience further sequelae of trauma, such as dissociative amnesia, resulting in the loss of personal memories or even their sense of identity. Anecdotal experiences of clinicians in Syrian hospitals, as witnessed by one of us (A.G.), indicate that numerous former prisoners exhibit pronounced dissociative symptoms and cognitive-perceptual disruptions. These include mistaking healthcare workers for prison guards and experiencing memory gaps, such as reporting their age as it was before incarceration. The assessment and management of these individuals are further complicated by the lack of collateral informants, such as known family members, who could help reconstruct an accurate timeline of a patient’s mental health condition.

Female detainees, in particular, suffered extremely harsh conditions in prisons, where several specific methods of torture were documented, including electrocuting or touching breasts, burning or beating genitalia and forced nudity. They were also deprived of feminine hygiene products. Additionally, there was a severe lack of prenatal and postnatal care, with several reported miscarriages and reports of self-inflicted abortions of fetuses conceived through rape.^5^ Reports have also revealed the existence of children born in prisons who were kept imprisoned with their mothers.

Rape has been used as a tool of dehumanisation and oppression, causing psychological harm. Children and adolescents were treated like adults, placed in the same cells and subjected to the same conditions and were also exposed to witnessing detainees being sexually abused, leaving them with unimaginable scars.^5^ Countless individuals remain missing, leaving families searching for closure. This collective trauma extends beyond the survivors, underscoring the urgency of addressing the psychological impact.

## Pathways to healing

Amid the profound psychological wounds inflicted by years of trauma, evidence-based strategies offer a pathway towards recovery, underscoring the importance of immediate and sustained care tailored to the unique needs of survivors.

First and foremost, it is essential to ensure a suitable living environment and initiate rehabilitation programmes for survivors and their families to help them reintegrate into normal social life and live with dignity. Equally important is attending to the medical needs of freed prisoners, many of whom have multiple health problems, either preceding their imprisonment or resulting from it. As a result, extensive medical follow-up by different specialties is often required, indicating the need for comprehensive support and coordinated care. Basic nutritional care is essential, and refeeding-related risks have already been highlighted in early medical reports. All of this must be part of the broader recovery of Syrian society and the state as a whole, addressing economic, security and service-related challenges.

Psychological first aid serves as a crucial first step in alleviating acute distress and reducing PTSD and other symptoms of psychological distress. It is important during this process to conduct a preliminary evaluation to identify cases that require follow-up, to focus efforts and achieve the best results, especially given the ongoing scarcity of resources in Syria. For individuals identified as needing further support, psychological first aid must be followed by continued care to address the long-term psychological impacts of traumatic experiences.^6,7^

Specialised therapies have proven effective in managing depression, suicidality, PTSD and related trauma symptoms. Approaches such as cognitive–behavioural therapy (CBT) for depression and anxiety, narrative exposure therapy (NET) and eye movement desensitisation and reprocessing (EMDR) for PTSD have shown significant success in helping survivors process and reframe traumatic memories.^8,9^

Internet-based therapies and brief psychoeducation sessions also provide accessible options for symptom management for people with depression, anxiety and other common mental disorders, particularly in resource-limited or conflict-affected settings.^10^ In addition to psychotherapy, medications remain essential tools in managing psychiatric symptoms and should be part of any comprehensive response.^11^ For cases of sexual violence, which are highly stigmatised and therefore difficult to reach, we recommend developing mechanisms that ensure privacy and build trust with survivors. Online interventions could be a viable solution. Awareness programmes should be initiated across society to reduce stigma related to all forms of mental illness, including but not limited to survivors of sexual violence.

Additionally, social support significantly influences the prognosis for PTSD,^12^ emphasising the need to reintegrate freed prisoners into their communities with the support of family, friends and mental health professionals. Providing immediate and sustained care is critical not only for the individual’s recovery but also to prevent long-term intergenerational psychosocial consequences of traumatic experiences.^13^

## A call to action

The mental health crisis among freed Syrian prisoners demands urgent attention and coordinated action. Delays in receiving clinical services for trauma survivors are significantly associated with higher rates of mental health symptoms, underscoring the importance of early intervention.^14^ To guide these efforts effectively, research is needed to assess the current mental health infrastructure and to establish prevalence data on psychiatric conditions among former detainees. A comprehensive, multi-tiered response is essential to address the scale and complexity of this crisis effectively.

Immediate interventions should prioritise outreach and screening efforts to identify survivors and provide psychological first aid and basic psychosocial support. Intermediate strategies must focus on building the capacity of local healthcare systems through training programmes for Syrian mental health providers in trauma-informed care and culturally adapted evidence-based therapies. For the long term, community-based mental health programmes and policy reforms should be implemented to ensure sustained support and recovery.

Local and international efforts must be coordinated to make mental health aid a key component of humanitarian assistance. Once the new authorities in Syria begin establishing a suitable working environment and basic infrastructure to ensure access to services for all segments of society, their active engagement will be crucial in coordinating efforts and securing international funding. Partnerships between global mental health organisations, Syrian non-governmental organisations (NGOs) and local official entities are necessary to integrate mental healthcare into rebuilding efforts.

Although this article focuses on Syria, comparisons with similar post-conflict contexts may offer valuable insights and should be explored in future work. Practical steps for individuals include donating to organisations supporting survivors, volunteering expertise, raising awareness and advocating for policy changes that prioritise mental health services. Local and international psychiatrists and mental healthcare providers are strongly encouraged to participate in this effort. This response must be guided by an understanding of the community’s cultural and social context, particularly when addressing sensitive issues such as sexual trauma.

The time to act is now. By combining outreach efforts with capacity building and global collaboration, we can help rebuild a healthcare system that offers freed Syrian prisoners a chance at healing and recovery.
